# Low Radioactivity Levels in Blood Samples After Targeted Radionuclide Therapy: Minimal Radiation Exposure of Healthcare Staff

**DOI:** 10.3390/biomedicines14030529

**Published:** 2026-02-27

**Authors:** Marcel Wehmann, Philipp Seifert, Christian Kühnel, Robert Drescher, Falk Gühne, Martin Freesmeyer

**Affiliations:** Clinic of Nuclear Medicine, Jena University Hospital, 07749 Jena, Germanyphilipp.seifert@med.uni-jena.de (P.S.); christian.kuehnel@med.uni-jena.de (C.K.); robert.drescher@med.uni-jena.de (R.D.); falk.guehne@med.uni-jena.de (F.G.)

**Keywords:** radioactivity, blood samples, I-131, Lu-177, radiation exposure, targeted radionuclide therapy

## Abstract

**Background/Objectives**: The increasing use of radiopharmaceuticals in clinical practice has raised concerns regarding potential radiation exposure for healthcare personnel handling biological samples from treated patients. The objective of this study was to assess the radioactivity levels in clinically necessary blood samples taken from patients treated with radioactive iodine-131 (I-131) or lutetium-177 (Lu-177) in a real-world setting. **Methods**: Prospective, tertiary care single-center study. Blood samples, at the clinically necessary time points, from 220 consecutive targeted radionuclide therapies (TRTs) used to treat 151 distinct patients between October 2021 and January 2025 were included. The influences of the eGFR and the time interval between tracer administration and blood sampling on radioactivity concentration were investigated by linear regression models. The applied amount of radioactivity was excluded as a confounder by adjusting all cases to 1 GBq. Statistical programming language R was utilized and *p* < 0.05 was considered significant. **Results**: The mean age of the patients was 62 years and 52% were male. Mean radioactivity concentrations of 6 vs. 60 kBq/mL were measured at 52 vs. 13 h after application of 1.9 vs. 6.7 GBq I-131 vs. Lu-177, respectively. Better renal function and later blood sampling were both associated with lower radioactivity concentration in blood samples (each *p* < 0.001). Total radioactivity levels in all samples were well below the upper limits for the disposal of biological samples (1 MBq for I-131 and 10 MBq for Lu-177). **Conclusions**: There was only a low exposure risk for nuclear medicine personnel and laboratory staff. These findings emphasize that handling blood samples from patients treated with I-131 and Lu-177 in clinical routine is minimal.

## 1. Introduction

The use of radiopharmaceuticals has become a cornerstone in the treatment of certain diseases, particularly in oncology and endocrinology. Radioactive iodine-131 (I-131) has long been a standard treatment modality for differentiated thyroid cancer (DTC) as well as for benign thyroid diseases (BTD) [1,2,3]. I-131 has a physical half-life of 8.0 days and undergoes radioactive decay with a characteristic photopeak at 364 keV [4,5].

In recent years, the development of lutetium-177 (Lu-177)-labeled compounds, particularly those targeting somatostatin receptors or prostate-specific membrane antigens (PSMA), has changed the management of neuroendocrine tumors (NET) and metastatic castration-resistant prostate cancer (mCRPC), leading to an increased number of targeted radionuclide therapies (TRTs) [6,7,8,9]. Lu-177 displays two main photopeaks, at 113 keV and 208 keV [10]. The physical half-life of Lu-177 is 6.7 days [11].

In accordance with German radiation protection regulations, TRTs must be carried out as an inpatient procedure [12]. Consequently, healthcare personnel are increasingly exposed to biologic samples from treated patients, including blood samples, which may contain radioactivity [13,14]. The transfer of these samples to departments outside of radiation protection areas, e.g., laboratories, is only permissible if it is in accordance with the established upper limits for disposal, which are 1 MBq [0.27 mCi] for I-131 and 10 MBq [2.7 mCi] for Lu-177 [15]. The safety of the medical staff, therefore, demands careful consideration, particularly in terms of potential radiation exposure during routine procedures [16].

Although there are studies that address radiation exposure based on whole-body dosimetry, these studies predominantly focus on nuclear medicine personnel [17,18]. In some studies, the radioactivity levels in blood samples are not directly measured but rather estimated from general physical models [19]. These models do not account for individual patient variability or the specific conditions under which blood samples were handled, which can lead to significant discrepancies between estimated and actual radioactivity levels in blood samples. For the most commonly used TRTs, I-131 and Lu-177, the number of real-world studies with actual measurements of the radioactivity levels in blood samples and subsequent analysis of the risk of exposure to medical personnel is limited [20,21]. Most existing studies have either estimated these values based on the administered dose of the radiopharmaceutical or focused solely on I-131, leaving a significant gap in the literature regarding other radionuclides such as Lu-177 [22].

The primary objective of this study was to prospectively generate empirical data on the actual radioactivity levels in clinically necessary blood samples taken from consecutive patients during routine clinical practice after they received therapeutic radiopharmaceuticals. We aimed to provide a robust foundation for estimating the exposure rates that medical staff may face when handling such samples, ultimately contributing to improved safety protocols. Emphasis was placed on the significance of renal function as a primary confounder in the context of residual radioactivity levels in blood samples.

## 2. Materials and Methods

### 2.1. Study Design, Ethics, and Patients

This prospective single-center study was conducted between October 2021 and January 2025 at a German tertiary care nuclear medicine department. The study was conducted in accordance with the Declaration of Helsinki and approved by the Institutional Ethics Committee (reference: 2022-2660-Material). Written informed consent was obtained from all participants or their legal guardians before inclusion in the study. All consecutive TRT cases from the following medical subgroups who required blood sample collection as part of their clinical management after TRTs were included in the study: firstly, patients with DTC after receiving radioactive iodine therapy (DTC-RAI) or undergoing diagnostic whole-body scintigraphy (DTC-WBS) and patients with BTD who received I-131 and, secondly, patients with a NET and mCRPC who received Lu-177. Products used were NaI-131-capsules (Curium, Burlington, VT, US), Lu-177-Pluvicto and Lu-177-Lutathera (both Novartis, Basel, Switzerland), Lu-177-PSMA-I&T, and Lu-177-DOTATOC (both in-house production). The respective treatment regimens required varying dosages. The estimated glomerular filtration rate (eGFR) was routinely determined in all patients on the day before treatment. The eGFR was calculated by the CKD-EPI formula, which is based on the gender, ethnicity, age and serum creatinine. All results are presented on a per-TRT basis and are not patient-specific.

### 2.2. Collected Data Variables and Patient Preparation

The following data was collected per TRT in Excel software (version 2408, Microsoft, Redmond, WA, USA): patient sex and age, eGFR, disease, applied tracer and radioactivity, radioactivity concentration in blood samples at the timepoint of blood sample collection, and duration of hospitalization. All DTC-RAI patients were treated during T3/T4 hormone withdrawal, while all DTC-WBS patients received recombinant TSH stimulation (Thyrogen^®^, Genzyme Transgenics Corp., Cambridge, MA, US). All NET cases were prepared with an amino acid infusion (LysaKare^®^ 25 g/25 g 1.000 mL over 2.5 h) immediately before Lu-177-DOTATOC was applied. No specific, standardized patient preparations were conducted before TRT for BTD and mCRPC patients.

### 2.3. Determination of Radioactivity Levels in Blood Samples

During the study period, blood samples were collected as part of the clinically indicated procedures. Time points of radioactivity therapy administration and of blood sampling were documented. For each patient, an additional blood sample in a 2.7 mL EDTA blood collection tube (S-Monovette^®^; Sarstedt, Nümbrecht, Germany) was taken for study purposes to assess the radioactivity level. The radioactivity levels were quantified by analyzing 0.5 mL of blood using a gamma counter (Isomed 2100, NuviaTech Healthcare, Dresden, Germany) calibrated for I-131 and Lu-177.

Subsequently, the measured radioactivity level was decay-corrected to the time the blood sample was taken, and radioactivity concentrations calculated for the total volume of the blood collection tube utilized at our facility (2.7 mL, EDTA) and for one milliliter. The measurements were conducted in a controlled laboratory setting, adhering to safety protocols and regulatory guidelines.

These measurements were then compared with European Union regulations for the disposal of biologic samples in order to facilitate the disposal of radionuclides [12,15].

### 2.4. Data Analysis and Statistics

Descriptive statistics were employed to summarize the demographic and clinical characteristics of the patient cohort. These data, which included means, median, standard deviations, range and percentages, were used to provide an overview of the cohort’s demographic and clinical characteristics.

Linear regression was used to analyze the influence of eGFR as well as time between tracer administration and blood sample (“time”) on radioactivity concentration for different tumor entities. Applied radioactivity was introduced as a confounder to adjust results for its influence on measured radioactivity concentration. To stabilize their variance, radioactivity concentration at the time of blood sampling and applied radioactivity values were log-transformed prior to linear regression. The applied radioactivity was identified as a significant confounder. Scaling the applied radioactivity by 1 GBq (regression standardization) resulted in the following models:

*log*(*radioactivity concentration*) = *β*_0_ + *β*_1_ × “*eGFR*” + *β*_2_ × *log*(*applied radioactivity/1 Gbq*) + *ε* *log*(*radioactivity concentration*) = *β*_0_ + *β*_1_ × “*time*” + *β*_2_ × *log*(*applied radioactivity/1 Gbq*) + *ε*.

Adjusting measured radioactivity for the effects of applied radioactivity:

*log*(*radioactivity concentration*) − *β*_2_ × *log*(*applied radioactivity/1 Gbq*) = *β*_0_ + *β*_1_ × “*eGFR*” *log*(*radioactivity concentration*) − *β*_2_ × *log*(*applied radioactivity/1 Gbq*) = *β*_0_ + *β*_1_ × “*time*”.

*p*-values are reported for the independent variables and eGFR as well as for time between tracer administration and blood sample, and *p* < 0.05 was considered significant [23]. Reported R^2^ values represent the proportion of variation in measured radioactivity explained by applied radioactivity and eGFR, as well as by time between tracer administration and blood sampling, respectively. The statistical programming language R was used for all computations (Version 4.5.2, R Development Core Team, Vienna, Austria).

## 3. Results

During the 40-month study period, the radioactivity levels in blood samples from the 220 TRTs (151 distinct patients) were measured and analyzed.

The gender ratio across all patients was 52.2% male and 47.7% female. The mean age of the patients was 61.7 ± 15.4 years. The analysis revealed that more than 79.6% of patients exhibited normal or minimally impaired renal function, with an eGFR greater than 60 mL/min.

Blood samples were analyzed using a gamma counter within 1–3 days after blood collection. Details of the entire cohort are presented in Table 1 for I-131 treatments and Table 2 for Lu-177-based therapies.

In accordance with German radiation protection regulations, all I-131 patients were hospitalized until the legal limit of 3.5 µSv/h was reached, for a minimum of 48 h [12]. For patients with Lu-177, the requirements for inpatient stays were determined by the responsible local authority. Patients with a NET and mCRPC normally stayed 48 h, in some cases 72 h for patient requiring individual dosimetry, and for logistical reasons. Individuals from the BTD subgroup exhibited the longest duration of hospitalization. In the groups in which TRT was administered due to an underlying malignant disease, there was considerable variation in the applied activity. Out of all subgroups, patients with DTC-RAI received the highest administered doses on average.

The intervals between radiopharmaceutical administration and blood sampling were variable, and were influenced by the duration of hospitalization. In consequence, intervals were longer in the BTD subgroup, and brief and relatively consistent in the Lu-177 subgroup.

The mean radioactivity concentration was found to be remarkably lower in patients receiving I-131 compared to those receiving Lu-177. The highest values were observed in patients with a NET, approximately three times the mean values of DTC-RAI patients, who recorded the second highest mean values. The measured values of the radioactivity concentration and the extrapolated values to the total radioactivity level of the entire blood collection tube used after administration of I-131 and Lu-177 are shown separately for each subgroup in Table 1 and Table 2.

**Figure 1:**
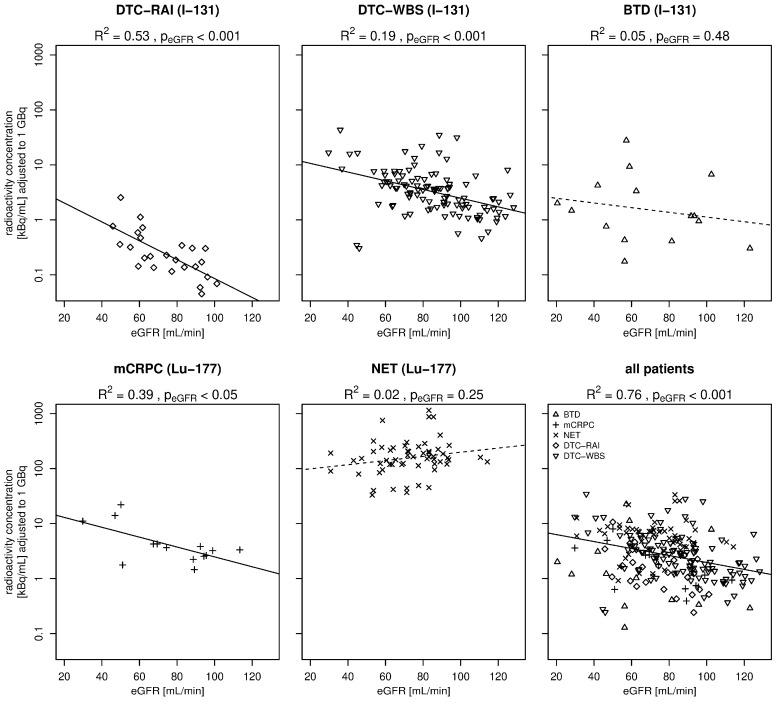
Radioactivity concentration at time of blood draw in relation to renal function (adjusted to 1 GBq). Abbreviations: DTC, differentiated thyroid cancer; RAI, radioactive iodine; WBS, whole-body scintigraphy; BTD, benign thyroid diseases; mCRPC, metastatic castration resistant prostate cancer; NET, neuroendocrine tumor; kBq/mL, kilobecquerel per milliliter; and eGFR, estimated glomerular filtration rate.

The analyses of renal function as a relevant influencing factor showed highly significant correlations between eGFR and radioactivity concentration across the entire cohort (*p* < 0.001 and R^2^ = 0.76); a higher eGFR correlated with lower radioactivity concentrations in the blood samples. This correlation was confirmed in most of the subgroups, except patients with BTD and a NET (Figure 1). Solid trend lines represent significant correlations and dashed trend lines represent non-significant results.

**Figure 2:**
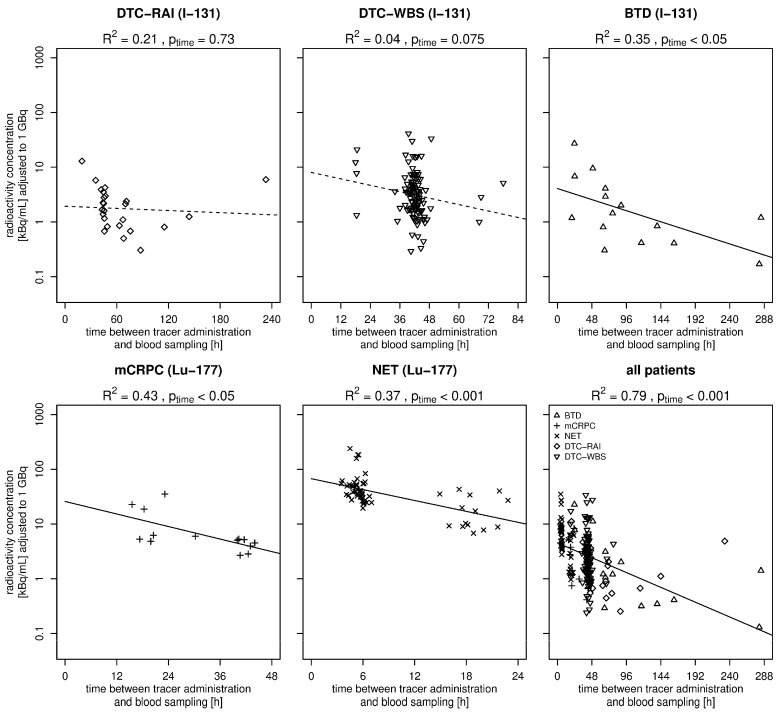
Radioactivity concentration in relation to the time between tracer administration and blood sampling (adjusted to 1 GBq). Abbreviations: DTC, differentiated thyroid cancer; RAI, radioactive iodine; WBS, whole-body scintigraphy; BTD, benign thyroid diseases; mCRPC, metastatic castration resistant prostate cancer; NET, neuroendocrine tumor; kBq/mL, kilobecquerel per milliliter.

The analyses of the time between tracer administration and blood sampling were determined as a relevant influencing factor and showed highly significant correlation with the measured radioactivity concentrations across the entire cohort (*p* < 0.001 and R^2^ = 0.79). Later time points of blood sampling strongly correlated with lower radioactivity concentrations in the blood samples. Significant correlations were confirmed in the subgroups BTD, mCRPC, and NET (Figure 2). Solid trend lines represent significant correlations and dashed trend lines represent non-significant results. The scatter plot at approximately 40 h for the DTC-WBS subgroup corresponds to the elective blood sampling used to determine the maximally stimulated tumor marker thyroglobulin in these patients. The scatter plot at approximately 6 h in the NET subgroup corresponds to the scheduled blood sampling for electrolyte analysis following amino acid and Lu-177-DOTATOC infusions.

## 4. Discussion

Analyzes of the blood samples collected after TRT revealed very low radioactivity levels in the blood collection tubes during the inpatient stay. In the I-131 patients, the highest mean radioactivity concentration was observed in the DTC-RAI subgroup (25.1 kBq/mL [0.7 µCi/mL]), which was recorded after a mean interval of 66 h after radiopharmaceutical administration. The mean level in a 2.7 mL blood collection tube was 64.9 kBq [1.8 µCi] in this group. Viagald-Miguel reported comparable values of up to 69 kBq [1.9 µCi] [21]. In comparison with the DTC-RAI group, patients in the DTC-WBS and BTD subgroups exhibited lower radioactivity levels in their blood samples, correlating to the considerably lower therapy activities administered. However, when adjusted to 1 GBq of therapy activity, the opposite was observable (Figure 1). In the DTC-RAI subgroup, following thyroidectomy, there was, frequently, minimal residual target tissue for the tracer, leading to its rapid elimination from the organ. The lower values for the DTC-RAI subgroup in comparison to the DTC-WBS subgroup may be attributable to the observation that the DTC-RAIs in our department were performed when patients were in hormone (T3/T4) withdrawal, while the DTC-WBS patients previously received Thyrogen. Furthermore, in the BTD subgroup, blood samples were taken at a later stage. Due to this time difference, it can be assumed that initial decay processes and tissue necrosis had already taken place, which could have caused I-131 isotopes to re-enter the bloodstream from the thyroid gland. This effect could have led to a relative increase in the measured blood activity and, thus, represents a possible compensating factor in comparison to the lower adjusted radioactivity levels in the DTC-RAI subgroup [24]. Mean radioactivity concentrations at the time of blood collection were 1.4 kBq/mL [0.04 µCi/mL] after a mean interval of 42 h for the DTC-WBS subgroup and 4.2 kBq/mL [0.1 µCi/mL] after a mean of 102 h for the BTD group, respectively. The values obtained in this study are comparable to previous studies. For instance, Larkin et al. reported values of 20 kBq/mL [0.54 µCi/mL] after 48 h and 10 kBq/mL [0.27 µCi/mL] after 72 h [20].

For the Lu-177-based therapies, radioactivity levels in the analyzed blood samples were found to be higher in the NET group than in the mCRPC group. This phenomenon can be attributed to the higher radioactivity applied to NET patients, and to the earlier timing of blood sampling. The latter were routinely performed in the NET group on the day of therapy as part of standardized electrolyte checks after amino acid infusion, as specified in the clinical protocol. Conversely, in the mCRPC group, the absence of elective blood samples following radioligand therapy leads to less-standardized measurement times. These structural differences in the clinical process resulted in the inclusion of a significantly higher number of NET patients in the analysis during the study period, despite the increased frequency of mCRPC treatments.

The maximum radioactivity level recorded in the blood collection tubes among all subjects treated with I-131 in our study was 222.4 kBq (=0.2 MBq [6.0 µCi]). Among all patients after administration of Lu-177, the highest measured value was that of a patient with a NET and amounted to 1009.2 kBq (=1.0 MBq [27.3 µCi]). All other values were found to be below these peak values and well below the European limits for the disposal of biologic samples (1 MBq [27.0 µCi] for I-131 and 10 MBq [270 µCi] for Lu-177), thereby permitting the transportation of these samples to other departments outside of the nuclear medicine unit, such as the laboratory, for further processing [15].

The resulting exposure rate provides a more meaningful understanding of the biological hazard for medical personnel handling the samples in comparison to the pure radioactivity levels. However, the estimation of the exposure rate is challenging and is based on numerous factors, such as time of handling, shielding by the wall of the blood collection tube and beta/gamma dose proportion. The following calculation of exposure was based on a worst-case scenario of holding the sample with the entire hand during processing, as well as the highest measured radioactivity level of the two nuclides used. The longest time period we observed of handling a blood collection tube at our laboratory was 32 s (because semiautomatic processing analyzes are utilized). A modified external dose equation was estimated to calculate the exposure based on those worst-case parameters (see Supplemental Methods) [25,26]. For the highest measured blood collection tube with the I-131, as determined by our team, this would result in a local hand exposure of 1.09 mSv. Subsequent to the application of Lu-177, an exposure of 4.04 mSv was ascertained for this theoretical case. Given the established annual exposure limits of 500 mSv to the skin of the hand, it is feasible to handle a maximum of 459 samples of the highest measuring I-131 blood collection tubes and 124 samples of the highest measuring Lu-177 blood collection tubes per year under these conservative assumptions [27,28]. While other studies report lower exposures they do not take into account our worst-case model [20,22,29]. Nevertheless, the expected radiation exposure risk for the personnel entrusted with the handling of blood samples is very low. When considering realistic conditions, it can be assumed that exposure levels would be markedly lower, given the substantial reduction in the beta radiation component at greater distances.

The radioactivity levels in the blood samples amongst all patients showed a statistically significant negative correlation with renal function since the urinary tract serves as the primary route of excretion for the radiopharmaceuticals under investigation [30,31,32]. A particularly strong correlation between the measured radioactivity levels in the blood and the eGFR was observed in the DTC-RAI and DTC-WBS subgroups. In both groups, there is typically very little or no remaining iodine-absorbing thyroid remnant or tumor tissue, so that only a minimal proportion of the tracer is taken up by the target tissue. The clearance of blood is primarily determined by renal excretion, thereby explaining the close correlation with renal function. In contrast, the other three subgroups exhibited a substantially higher concentration of tracer-absorbing target tissue, which contributes to clearance mechanisms beyond renal excretion. Among these, a substantial correlation with eGFR was observed exclusively in the mCRPC subgroup. However, this subgroup comprised the smallest cohort in the study (*N* = 14). The most targeted tissue is present in the BTD subgroup, wherein tracer uptake by the thyroid gland is typically 30–60% [33]. Consequently, renal function exhibited no substantial influence on the radioactivity levels within this very limited cohort (*N* = 15).

The collection of urine samples following the administration of the TRT was not incorporated into the study’s design, as this procedure did not constitute a standard component of any clinical treatment regimen. The process of excretion results in an accumulation of radioactivity in the urine. The radioactivity level in the urine samples would be expected to be many times higher and could approach or even exceed the existing regulatory limits. However, urine samples are rarely necessary in routine clinical settings. One example of this phenomenon would be a suspected urinary tract infection. In order to avoid unnecessary exposure, any symptoms should be specifically queried so that urine samples can be taken before the TRT is applied. In the future, the potential value of urine samples in determining the presence of radioactivity should be considered, as there is a paucity of literature on this subject.

The present study lacks information regarding the value of dosimetry in providing an effective imaging approach to calculate absorbed doses for target and non-target organs. Future studies should focus on providing effective measurements for the in vivo blood activity of TRT agents using both biochemical and imaging-based indices.

A notable limitation of this study is the considerable variability in the size of the individual subgroups, because only blood samples taken for clinical reasons were examined. For the patients with a NET, blood samples were obtained in a standardized manner to assess serum potassium levels following the administration of an amino acid solution (nephroprotection). In the DTC-WBS subgroup, blood samples were electively drawn in order to measure the maximum level of thyroglobulin (tumor marker) after stimulation with recombinant TSH (Thyrogen^®^). Conversely, the treatment of DTC-RAI patients at this clinic typically involves hormone withdrawal, thereby obviating the need for additional blood samples subsequent to therapy. In all other instances, blood samples were only collected for the management of clinical symptoms, such as in cases where infection was suspected during hospitalization.

Examining the cohort reveals significant heterogeneity in the prescribed therapy activities in the individual subgroups and the corresponding times of blood collection after administration. This heterogeneity stems from the study design, which aimed to reflect real-world data. However, these aspects also represent important limitations. Especially, the temporal variability in blood sample collection represents a significant confounding factor, leading to the introduction of temporal bias.

Because time is a significant factor in determining the radioactivity levels of nuclides, future studies should implement a prospective standardized measurement schedule and uniform sampling strategy to improve the comparability of measured radioactivity levels between different subgroups.

## 5. Conclusions

This study provides empirical real-world data on the radioactivity levels in blood samples following TRT with I-131 and Lu-177. The results show that radiation exposure for healthcare professionals when handling these samples is low. The study underscores the significance of renal function in influencing radioactivity levels, highlighting its crucial role in the management of radioactive waste and the safety of medical staff. It is recommended that future studies be conducted to enhance the generalizability and reliability of these findings. This may be achieved by expanding the sample size and implementing prospective standardized blood sampling protocols.

## Figures and Tables

**Table 1:** Data of patients receiving I-131.

Patient Data and Treatment Parameters	All I-131 Patients (*N* = 148)	DTC-RAI (*N* = 25)	DTC-WBS (*N* = 108)	BTD (*N* = 15)
Male	61 (31%)	17 (68%)	36 (33%)	8 (53%)
Female	87 (59%)	8 (32%)	72 (67%)	7 (47%)
Age [years]	58.9 ± 17.3; 61.5 (19.0–90.0)	67.7 ± 11.9; 68.0 (35.0–84.0)	55.6 ± 17.3; 58.0 (20.0–91.0)	67.6 ± 15.7; 73.0 (26.0–83.0)
eGFR [mL/min]	81.4 ± 22.6; 82.7 (20.5–128.2)	73.8 ± 16.6; 74.4 (45.8–101.2)	85.1 ± 21.8; 86.1 (29.7–128.2)	67.7 ± 28.1; 58.9 (20.5–123.0)
>90	57 (39%)	6 (24%)	46 (43%)	5 (33%)
60–90	64 (43%)	13 (52%)	49 (45%)	2 (13%)
30–59	24 (16%)	6 (24%)	12 (11%)	6 (41%)
<30	3 (2%)	0	1 (1%)	2 (13%)
Duration of hospitalization [h]	84.0 ± 55.2; 72.0 (48.0–312.0)	118.1 ± 47.0; 96.0 (72.0–288.0)	62.4 ± 11.7; 72.0 (48.0–72.0)	193.6 ± 91.7; 192.0 (72.0–312.0)
Applied radioactivity [GBq]	1.9 ± 3.2; 0.4 (0.3–13.0)	8.6 ± 2.8; 9.0 (3.7–13.0)	0.4 ± 0.02; 0.4 (0.3–0.5)	1.4 ± 0.8; 1.1 (0.6–3.5)
Time between tracer administration and blood sampling [h]	52.0 ± 37.0; 43.0 (18.0–283.5)	65.5 ± 42.8; 46.3 (19.6–233.1)	42.0 ± 7.2; 42.0 (18.0–78.0)	101.9 ± 80.9; 66.8 (20.0–283.5)
radioactivity concentration [kBq/mL] at the time of blood sampling	5.7 ± 13.0; 1.1 (0.1–82.4)	25.1 ± 22.3; 18.7 (2.2–82.4)	1.4 ± 1.8; 0.9 (0.1–11.5)	4.2 ± 7.2; 1.3 (0.2–29.5)
total amount of radioactivity at the time of blood sampling [kBq], extrapolated to 2.7 mL	15.6 ± 34.3; 2.9 (0.2–222.4)	64.9 ± 59.2; 41.9 (5.9–222.4)	4.8 ± 8.7; 2.4 (0.2–56.4)	11.1 ± 18.7; 3.3 (0.5–76.7)

Data are shown as mean ± SD; median (min–max) or number (%) of patients. Abbreviations: DTC, differentiated thyroid cancer; RAI, radioactive iodine; WBS, whole-body scintigraphy; BTD, benign thyroid diseases; and eGFR, estimated glomerular filtration rate.

**Table 2:** Data of patients receiving Lu-177.

Patient Data and Treatment Parameters	All Lu-177 Patients (*N* = 72)	mCRPC (*N* = 14)	NET (*N* = 58)
Male	54 (75%)	14 (100%)	40 (69%)
Female	18 (25%)	0	18 (31%)
Age [years]	67.5 ± 8.2; 69 (45.0–81.0)	68.6 ± 8.5; 68.0 (56.0–81.0)	67.3 ± 8.1; 69.0 (45.0–80.0)
eGFR [mL/min]	73.3 ± 18.5; 73.4 (29.8–114.2)	75.8 ± 23.4; 81.5 (29.8–113.4)	72.7 ± 17.0; 72.1 (30.8–114.2)
>90	12 (17%)	5 (36%)	7 (12%)
60–90	42 (58%)	5 (36%)	37 (64%)
30–59	17 (24)	3 (21%)	14 (24%)
<30	1 (1%)	1 (7%)	0
Duration of hospitalization [h]	48.0 ± 0.0; 48.0 (48.0–48.0)	49.7 ± 6.2; 48.0 (48.0–72.0)	49.7 ± 6.1; 48.0 (48.0–72.0)
Applied radioactivity [GBq]	6.7 ± 1.4; 7.4 (2.1–10.2)	5.4 ± 1.9; 5.2 (2.1–8.0)	7.0 ± 1.1; 7.5 (4.1–10.2)
Time between tracer administration and blood sampling [h]	13.0 ± 11.4; 6.1 (3.5–44.0)	31.2 ± 11.0; 35.1 (15.6–44.0)	8.6 ± 5.8; 5.9 (3.5–22.8)
radioactivity concentration [kBq/mL] at the time of blood sampling	59.8 ± 65.0; 49.2 (4.4–373.8)	14.4 ± 14.5; 7.9 (4.4–54.8)	70.8 ± 67.6; 53.9; (10.6–373.8)
total amount of radioactivity at the time of blood sampling [kBq], extrapolated to 2.7 mL	159.4 ± 175.2; 128.9 (11.5–1009.2)	43.7 ± 51.0; 20.4 (11.5–178.5)	187.3 ± 183.0; 139.2 (27.6–1009.2)

Data are shown as mean ± SD; median (min–max) or number (%) of patients. Abbreviations: mCRPC, metastatic castration resistant prostate cancer; NET, neuroendocrine tumor; and eGFR, estimated glomerular filtration rate.

## Data Availability

The original contributions presented in this study are included in the article/Supplementary Material. Further inquiries can be directed to the corresponding author.

## Supplementary Materials

The following supporting information can be downloaded at: https://www.mdpi.com/article/10.3390/biomedicines14030529/s1, mathematical component1: modified external dose equation.

## Abbreviations

The following abbreviations are used in this manuscript:

BTD	benign thyroid diseases
CKD-EPI	Chronic Kidney Disease Epidemiology Collaboration
DTC	differentiated thyroid cancer
DTC-RAI	differentiated thyroid cancer after radioactive iodine therapy
DTC-WBS	differentiated thyroid cancer after diagnostic whole-body scintigraphy
eGFR	estimated glomerular filtration rate
EU	European Union
GBq	gigabecquerel
I-131	iodine-131
kBq	kilobecquerel
keV	kiloelectronvolt
Lu-177	lutetium-177
MBq	megabecquerel
mCi	millicurie
mCRPC	metastatic castration-resistant prostate cancer
mL	milliliter
µSv/h	microsievert per hour
NET	neuroendocrine tumors
PSMA	prostate-specific membrane antigen
RAI	radioactive iodine therapy
TRT	targeted radionuclide therapy
TSH	thyroid-stimulating hormone

## References

[1] Luster M., Clarke S.E., Dietlein M., Lassmann M., Lind P., Oyen W.J., Tennvall J., Bombardieri E. (2008). Guidelines for radioiodine therapy of differentiated thyroid cancer. Eur. J. Nucl. Med. Mol. Imaging.

[2] Unlu M.T., Kostek M., Aygun N., Isgor A., Uludag M. (2022). Non-Toxic Multinodular Goiter: From Etiopathogenesis to Treatment. Med. Bull. Sisli Etfal Hosp..

[3] Weetman A.P. (2007). Radioiodine treatment for benign thyroid diseases. Clin. Endocrinol..

[4] Burkinshaw L. (1958). The half-life of iodine 131. Phys. Med. Biol..

[5] Krajewska G., Pachocki K.A. (2013). Assessment of exposure of workers to ionizing radiation from radioiodine and technetium in nuclear medicine departmental facilities. Med. Pr..

[6] Strosberg J., El-Haddad G., Wolin E., Hendifar A., Yao J., Chasen B., Mittra E., Kunz P.L., Kulke M.H., Jacene H. (2017). Phase 3 Trial of ^177^Lu-Dotatate for Midgut Neuroendocrine Tumors. N. Engl. J. Med..

[7] Sartor O., de Bono J., Chi K.N., Fizazi K., Herrmann K., Rahbar K., Tagawa S.T., Nordquist L.T., Vaishampayan N., El-Haddad G. (2021). Lutetium-177-PSMA-617 for Metastatic Castration-Resistant Prostate Cancer. N. Engl. J. Med..

[8] Schutze C., Borowski M., Freesmeyer M., Freudenberg R., Hanscheid H., Kurth J., Linde M.R., Militzer L.C., Sattler B., Schmidt T.N. (2026). ^177^Lu-based radioligand therapy: A retrospective multicenter analysis to calculate the effective half-life and follow-up dose for the public. Nuklearmedizin-NuclearMedicine.

[9] World Nuclear Association Radioisotopes in Medicine. https://world-nuclear.org/information-library/non-power-nuclear-applications/radioisotopes-research/radioisotopes-in-medicine.

[10] Huizing D.M.V., Sinaasappel M., Dekker M.C., Stokkel M.P.M., de Wit-van der Veen B.J. (2020). ^177^Lutetium SPECT/CT: Evaluation of collimator, photopeak and scatter correction. J. Appl. Clin. Med. Phys..

[11] Pillai A.M., Knapp F.F. (2015). Evolving Important Role of Lutetium-177 for Therapeutic Nuclear Medicine. Curr. Radiopharm..

[12] Regulation on Protection Against the Harmful Effects of Ionizing Radiation (Radiation Protection Regulation) (BGBl I 41/2018). https://www.verwaltungsvorschriften-im-internet.de/bsvwvbund_17102011_RSII4114321.htm.

[13] Sunny S.S., Hephzibah J., Mathew D., Bondu J.D., Shanthly N., Oommen R. (2018). Stimulated Serum Thyroglobulin Levels versus Unstimulated Serum Thyroglobulin in the Follow-up of Patients with Papillary Thyroid Carcinoma. World J. Nucl. Med..

[14] Lapa C., Werner R.A., Bluemel C., Luckerath K., Schirbel A., Strate A., Buck A.K., Herrmann K. (2014). Influence of the amount of co-infused amino acids on post-therapeutic potassium levels in peptide receptor radionuclide therapy. EJNMMI Res..

[15] Council of the European Union (2014). Council Directive 2013/59/Euratom of 5 December 2013 Laying Down Basic Safety Standards for Protection Against the Dangers Arising from Exposure to Ionising Radiation, and Repealing Directives 89/618/Euratom, 90/641/Euratom, 96/29/Euratom, 97/43/Euratom and 2003/122/Euratom.

[16] Schwartz J.G., Phillips W.T., Shriver C.A., Okorodudu A.O. (1991). The Risk of Radiation Exposure to Laboratory Personnel. Lab. Med..

[17] Alkhorayef M., Sulieman A., Mohamed-Ahmed M., Al-Mohammed H.I., Alkhomashi N., Sam A.K., Bradley D.A. (2018). Staff and ambient radiation dose resulting from therapeutic nuclear medicine procedures. Appl. Radiat. Isot..

[18] Piwowarska-Bilska H., Birkenfeld B., Gwardys A., Supinska A., Listewnik M.H., Elbl B., Cichon-Bankowska K. (2011). Occupational exposure at the Department of Nuclear Medicine as a work environment: A 19-year follow-up. Pol. J. Radiol..

[19] Suciu C.G., Amurao M., Luechtefeld D., Marko A., Ashby L., Gronowski A.M. (2021). Evaluation of Radioactivity in Patient Specimens Received in the Core Laboratory at a National Cancer Institute (NCI) Designated Cancer Center. Clin. Chem..

[20] Larkin A., Millan E., Noz M., Wagner S., Friedman K., Blum M. (2011). Radioactivity of blood samples taken from thyroidectomized thyroid carcinoma patients after therapy with (131)I. Thyroid.

[21] Vialard-Miguel J., Georges A., Mazere J., Ducassou D., Corcuff J.B. (2005). 131I in blood samples: A danger for professionals? A problem for immunoassays?. J. Nucl. Med. Technol..

[22] Sarah T.B., Peiling Y., Loo T.P., Ming W.Y., Yien L.S., Soon T.Y., Loke K.S.H. (2021). Radiation exposure to allied health personnel handling blood specimens from patients receiving radioactive iodine-131 and recombinant human TSH (thyrogen(R)) stimulation. J. Radiol. Prot..

[23] Di Leo G., Sardanelli F. (2020). Statistical significance: P value, 0.05 threshold, and applications to radiomics-reasons for a conservative approach. Eur. Radiol. Exp..

[24] Bonnema S.J., Hegedus L. (2012). Radioiodine therapy in benign thyroid diseases: Effects, side effects, and factors affecting therapeutic outcome. Endocr. Rev..

[25] Bourgois L., Menard S., Comte N. (2017). Calculation of Skin Dose Due to Beta Contamination Using the New Quantity of the Icrp 116: The ‘Local Skin Dose’. Radiat. Prot. Dosim..

[26] Peplow D.E. (2020). Specific Gamma-Ray Dose Constants with Current Emission Data. Health Phys..

[27] International Atomic Energy Agency (2014). Radiation Protection and Safety of Radiation Sources: International Basic Safety Standards (2014).

[28] Wrixon A.D. (2008). New ICRP recommendations. J. Radiol. Prot..

[29] Kuhnel C., Winkens T., Seifert P., Drescher R., Freesmeyer M. (2018). Radiation Exposure of the Investigator during Navigated Fusion of ^124^iodine Pet Imaging and Ultrasound. Radiat. Prot. Dosim..

[30] Vogel K., Opfermann T., Wiegand S., BIermann J., Busch M., Winkens T., Freesmeyer M. (2013). Relationship between estimated glomerular filtration rate and biological half-life of 131I. Nuklearmedizin.

[31] Kurth J., Krause B.J., Schwarzenbock S.M., Stegger L., Schafers M., Rahbar K. (2018). External radiation exposure, excretion, and effective half-life in ^177^Lu-PSMA-targeted therapies. EJNMMI Res..

[32] Yordanova A., Eppard E., Kurpig S., Bundschuh R.A., Schonberger S., Gonzalez-Carmona M., Feldmann G., Ahmadzadehfar H., Essler M. (2017). Theranostics in nuclear medicine practice. Onco Targets Ther..

[33] Kobe C., Eschner W., Wild M., Rahlff I., Sudbrock F., Schmidt M., Dietlein M., Schicha H. (2010). Radioiodine therapy of benign thyroid disorders: What are the effective thyroidal half-life and uptake of 131I?. Nucl. Med. Commun..

