# Supplementary material for: Low Radioactivity Levels in Blood Samples After Targeted Radionuclide Therapy: Minimal Radiation Exposure of Healthcare Staff
Source: Biomedicines. 2026 Feb 27;14(3):529. doi: 10.3390/biomedicines14030529 (PMC13024209; doi:10.3390/biomedicines14030529)
Supplement: Supplementary file 1 [file biomedicines-14-00529-s001.zip › Supplemental Methods.pdf]

Dose estimation for beta and gamma dose proportion [1, 2]

Estimating the radiation dose from a nuclear medicine human specimen is not straightforward, as both beta and gamma emissions must be considered.

$$\dot{D}_{overall} = \dot{D}_{contact;\,\beta} + \dot{D}_{effectiv;\,\gamma}$$

Beta particles primarily contribute to local skin dose due to their limited range, while gamma radiation can penetrate tissue and cause exposure at a distance. The resulting dose depends on several factors, including activity, exposure time, distance, and shielding, making accurate dose assessment complex which should be treated with caution.

For a simple estimation of the beta component, the local skin absorbed dose (LSD) values published by Bourgois et al. (2017) for I-131 (1.10E-06 Sv/h per Bq) and Lu-177 (9.00E-07 Sv/h per Bq) can be used with an transmission factor „T" for beta radiation with maximum energy E through material thickness z, which takes into account the weakening of the polypropylene (PP) density (0,9 g/cm^3) in the wall of the monovette.

$$\dot{D}_{contact,\beta} = LSD * A * t * T(z)$$

As both radionuclides attain comparable beta range maximum values of 1.9 and 2 mm in tissue, T(z) was assumed as 0.5.

$$T(z) = 1 - \frac{z}{R_{max}} \approx 0{,}5$$

For gamma radiation, a conservative dose estimation can be made using the point source formula based on the inverse square law. This approach assumes isotropic emission and full 4π exposure geometry, which is appropriate for close-contact scenarios such as handling radioactive specimens. To estimate the effective dose, the absorbed dose is multiplied by a radiation weighting factor (W_R) of 1 for photons and a tissue weighting factor (W_T) of 0.01 for skin, in accordance with ICRP recommendations. This yields a simplified yet protective estimation of the effective dose to the hands or other superficial tissues.

$$\dot{D}_{effectiv;\,\gamma} = \dot{D}_{absorbed,;\gamma} * W_{\text{radiation weighting factors}} * W_{Tissue}$$

The updated gamma dose rate constants for I-131 (5.468E−5 mSv·h^{-1}·MBq^{-1}) and Lu-177 (4.918E−6 mSv·h^{-1}·MBq^{-1}) measured at 100 cm published by Peplow et al. (2020) provide radionuclide-specific values that reflect refined physical decay data and improved computational models can be used. For

$$\dot{D}_{absorbed,;\gamma} = \frac{A * \Gamma * \mathrm{t}}{r^2}$$

It is essential that activity 'A' and processing time "t" are assumed to be at a distance 'r' of 0.1 cm, as above. It is obvious that the attenuation effect for the gamma photons of both radionuclides is almost 1% and therefore negligible.

References:

1. Bourgois L, Menard S, Comte N. Calculation of Skin Dose Due to Beta Contamination Using the New Quantity of the Icrp 116: The 'Local Skin Dose'. *Radiat Prot Dosimetry*. 2017;176:365-379.
2. Peplow DE. Specific Gamma-Ray Dose Constants with Current Emission Data. *Health Phys*. 2020;118:402-416.
